# Isoviolanthin Extracted from *Dendrobium officinale* Reverses TGF-β1-Mediated Epithelial–Mesenchymal Transition in Hepatocellular Carcinoma Cells via Deactivating the TGF-β/Smad and PI3K/Akt/mTOR Signaling Pathways

**DOI:** 10.3390/ijms19061556

**Published:** 2018-05-23

**Authors:** Shangping Xing, Wenxia Yu, Xiaofeng Zhang, Yingyi Luo, Zhouxi Lei, Dandan Huang, Ji Lin, Yuechun Huang, Shaowei Huang, Feifei Nong, Chunhua Zhou, Gang Wei

**Affiliations:** 1 School of Pharmaceutical Science, Guangzhou University of Chinese Medicine, Guangzhou Higher Education Mega Center, Guangzhou 510006, China; shopingxing@gmail.com (S.X.); yuwenxia3486@outlook.com (W.Y.); zhangxiaofeng3486@outlook.com (X.Z.); luoyingyi3486@outlook.com (Y.L.); leizhouxi@outlook.com (Z.L.); dandan3486@outlook.com (D.H); linji88@gzucm.edu.cn (J.L.); shaoweihuang021@outlook.com (S.H.); feifeinong@outlook.com (F.N.); chunhuazhou021@outlook.com (C.Z.); 2 The First Affiliated Hospital of Guangzhou University of Chinese Medicine, Guangzhou 510006, China; huangyuechun3486@outlook.com

**Keywords:** *Dendrobium officinale*, isoviolanthin, structural identification, epithelial–mesenchymal transition (EMT), TGF-β/Smad, PI3K/Akt/mTOR, hepatocellular carcinoma (HCC) cells

## Abstract

*Dendrobium officinale* is a precious medicinal herb and health food, and its pharmacological actions have been studied and proved. However, the mechanisms by which its active flavonoid glycosides affect epithelial–mesenchymal transition (EMT) in hepatocellular carcinoma (HCC) cells, such as HepG2 and Bel-7402 cells, have not been previously investigated. Therefore, we investigated whether isoviolanthin extracted from the leaves of *Dendrobium officinale* inhibits transforming growth factor (TGF)-β1-induced EMT in HCC cells. In this study, the physicochemical properties and structure of isoviolanthin were identified by HPLC, UV, ESIMS, and NMR and were compared with literature data. HCC cells were pretreated with 10 ng/mL TGF-β1 to induce EMT and then treated with isoviolanthin. Herein, we found that isoviolanthin exhibited no cytotoxic effects on normal liver LO2 cells but notably reduced the migratory and invasive capacities of TGF-β1-treated HCC cells. Additionally, isoviolanthin treatment decreased matrix metalloproteinase (MMP)-2 and -9 levels, and remarkably altered the expression of EMT markers via regulating the TGF-β/Smad and PI3K/Akt/mTOR signaling pathways; Western blot analysis confirmed that the effects of the inhibitors SB431542 and LY294002 were consistent with those of isoviolanthin. These findings demonstrate the potential of isoviolanthin as a therapeutic agent for the treatment of advanced-stage metastatic HCC.

## 1. Introduction

Hepatocellular carcinoma (HCC), a primary malignancy of the liver, is the most common type of cancer and now the third-leading cause of cancer death worldwide [1]. The major reported causes of HCC are Hepatitis B virus (HBV)and Hepatitis C virus (HCV)infection, and the incidence and mortality rates of HCC have been increasing in both sexes in East/Southeast Asia, Northern and Central Europe, and the USA over the past decades [2,3]. Although liver resection is still an effective and important treatment of choice for patients with HCC, it is feasible in only a small minority of early-stage cases [4]. Despite the initial success of curative strategies, including chemotherapeutic drugs and liver transplantation, severe toxic side effects and the distant metastasis of advanced-stage HCC account for the majority of HCC-related deaths [5,6]. Therefore, the identification of potent, nontoxic antimetastasis drugs will play a crucial role in the treatment of HCC.

Accumulating evidence suggests that epithelial–mesenchymal transition (EMT) is a key initiating step in the metastasis and invasion of cancer [7,8,9]. During EMT, cancer cells lose their intercellular tight junctions and epithelial characteristics, such as ZO-1 and E-cadherin expression, and acquire mesenchymal traits, including vimentin, N-cadherin, and fibronectin expression. Transforming growth factor (TGF)-β1 is one of the most potent inducers of EMT, and it promotes HCC progression and metastasis [10,11]. Matrix metalloproteinases (MMPs) have been reported to play an essential role in tumor progression that could promote tumorigenesis and stimulate EMT signaling, and MMP-2 and -9 are commonly upregulated in several types of cancer, including HCC [12,13]. TGF-β1 binds to two cognate serine–threonine kinase receptors on the cancer cell surface; the type I receptor TβRII transphosphorylates the type I receptor TβRI, leading to the phosphorylation of R-Smads that form complexes with co-Smads in the nucleus, and these complexes bind DNA to regulate EMT target genes via cooperative interactions with Slug, Snail, and other transcription factors [14]. Many studies have shown that TGF-β1 also activates non-Smad signaling pathways, including the MAPK, Rac1, and phosphatidylinositol-3-kinase (PI3K)/protein kinase B (Akt)/mammalian target of rapamycin (mTOR) pathways, which are known to be involved in TGF-β1-mediated EMT [15,16,17,18]. In particular, the PI3K/Akt/mTOR signaling pathway has been reported to play an essential role in regulating EMT [19,20].

*Dendrobium officinale*, a perennial epiphytic herb in the *Orchidaceae* family, grows mainly in China, the United States, Australia, and Japan. A variety of *Dendrobium officinale* extracts and polysaccharides have been proven to possess excellent anticancer activity in many different types of cancer, including in vitro cancer models such as HepG2, HCT-116, HelaS3, and CNEl cells and in vivo models such as the mouse Lewis lung carcinoma and rat gastric carcinoma models [21,22,23,24]. However, most of the anticancer reports on *Dendrobium officinale* are related to its polysaccharides and extracts; isolation and identification of a single flavonoid glycoside are rarely reported. Isoviolanthin, a flavonoid glycoside, was previously found in *Glycyrrhiza* species by Yang et al. [25], and it can also be isolated from other traditional medicinal herbs, such as *Dendrobium officinale*, *Dendrobium crystallinum*, and *Viola etrusca* [26,27,28]. However, none of the previous research has focused on the pharmacological effects of isoviolanthin, perhaps because the amount of isoviolanthin extracted is insufficient to carry out experiments.

In this study, isoviolanthin was extracted from *Dendrobium officinale* leaves, and its structure was identified by ESIMS, UV, ^1^H-NMR, and ^13^C-NMR spectra and compared with previous literature data. Given that 10 ng/mL TGF-β1 can activate both the TGF-β/Smad and PI3K/Akt/mTOR pathways to promote EMT in cancer cells, we hypothesized that deactivating these two signaling pathways may reverse EMT in TGF-β-treated HCC cells. Therefore, we investigated whether isoviolanthin abrogates EMT, invasion, and migration induced by TGF-β1 in HCC cells. Finally, the results highlighted that isoviolanthin could markedly inhibit TGF-β1-mediated migration and invasion by deactivating EMT via the TGF-β/Smad and PI3K/Akt/mTOR pathways, indicating that isoviolanthin may be a potential anticancer agent for the treatment of metastatic HCC.

## 2. Results

### 2.1. Structural Identification of Isolated Isoviolanthin

**Figure 1:**
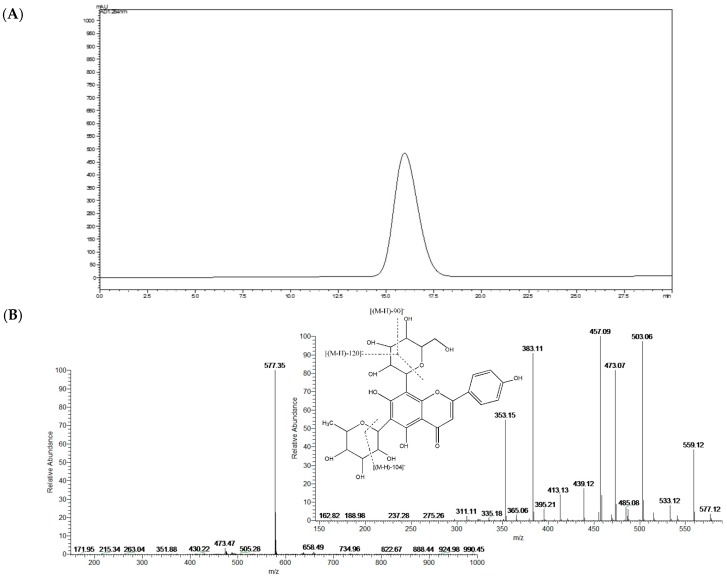
Structural identification of isoviolanthin. (**A**) The HPLC chromatographic analysis and (**B**) MS and MS^2^ spectra of isoviolanthin.

The compound isolated from the leaves of *Dendrobium officinale* was yellow and could be dissolved in methanol and water. The compound turned fuchsia after reaction with magnesite powder and HCl, which indicated that the compound was a flavonoid. In addition, the results of the Molisch reaction were positive, which confirmed that the compound was a flavonoid glycoside. The chemical reaction test also indicated that the compound was a flavonoid glycoside. UV experiments revealed absorption peaks at 216 nm, 271 nm, and 336 nm, similar to the characteristic absorption of flavonoids in the presence of UV. The purity of the compound was greater than 98% as determined by HPLC (Figure 1A). The ESIMS results showed that the *m*/*z* of the compound was 577 [M-H]^−^; therefore, the molecular weight of the compound was 578, and the molecular formula of the compound was C_27_H_30_O_14_, which was previously described in *Dendrobium officinale* by Ye et al. [26]. The MS and MS^2^ spectra of C_27_H_30_O_14_ are shown in Figure 1B.

The chemical shift in the ^1^H-NMR and ^13^C-NMR of the compound were as follows: ^1^H NMR (400 MHz, DMSO): δ: 13.91 (s, 1H), 8.03 (d, *J* = 8.8 Hz, 2H), 6.90 (d, *J* = 8.8 Hz, 2H), 6.80 (s, 1H), 5.02 (d, *J* = 5.8 Hz, 1H), 4.71 (d, *J* = 10.1 Hz, 1H), 1.26 (d, *J* = 5.2 Hz, 3H). ^13^C-NMR (101 MHz, DMSO): δ: 162.69, 102.82, 182.64, 161.66, 107.59 (C-6), 164.52, 105.44, 155.54, 103.58, 122.03, 129.47, 116.31, 157.65, 116.31, 129.47, 73.80, 71.18, 79.18, 71.18, 82.32, 61.85, 74.90, 72.23, 74.55, 72.64, 77.73, 18.67.

In the ^1^H-NMR spectra, many of the important signals of flavonoids were found. The single peak at δ 13.91 can be attributed to the hydroxyl hydrogen in position 5 of the A ring of the flavonoid. The single peaks in the lower fields at δ 6.80 can be attributed to position 3 in the B ring of the flavonoid. The signals at δ 8.03 and δ 6.90 are two dd peaks, which can be attributed to the 2′, 3′ and 5′, 6′ positions in the B ring of the flavonoid. The signals at δ 5.02 and 4.71 are the two signals for the anomeric positions of the sugar. The signal at δ 1.26 is from the hydrogen in the methyl group. Comparison of the compound spectral data with literature data proved it to be isoviolanthin. The ^1^H-NMR and ^13^C-NMR spectra of isoviolanthin are shown in Supplementary Materials Figures S1 and S2.

### 2.2. The Effect of Isoviolanthin on the Growth of Hepatocellular Carcinoma (HCC) and LO2 Cells

**Figure 2:**
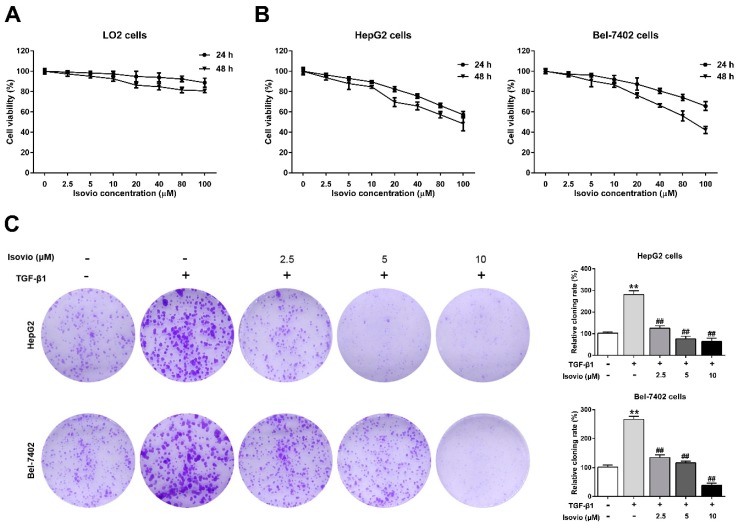
Effects of isoviolanthin (isovio) on the viability of hepatocellular carcinoma (HCC) and human LO2 normal liver cells and on the clonogenic potential of HCC cells. (**A**) The cell viability of LO2; (**B**) HepG2, and Bel-7402 cells treated with isoviolanthin (2.5 µM, 5 µM, 10 µM, 20 µM, 40 µM, 80 µM, and 100 μM) for 24 h and 48 h was determined by MTT [3-(4,5-dimethythiazol-2-yl)-2,5-diphenyl tetrazolium bromide] assay; (**C**) HepG2 and Bel-7402 cells were incubated with 10 ng/mL transforming growth factor (TGF)-β1 and isoviolanthin (2.5 µM, 5 µM, and 10 µM) for two weeks, and the clonogenic potential of each group was analyzed by colony formation assay. Representative images and bar graphs (mean ± SD) are shown, *n* = 3. ** *p* < 0.01 versus the control group; ^##^ *p* < 0.01 versus the TGF-β1 group.

To determine the optimal dose of isoviolanthin to reverse EMT in HCC cells without negatively affecting proliferation in the EMT experiments, we assessed the viability of HepG2, Bel-7402, and normal hepatic LO2 cells treated with isoviolanthin (2.5 µM, 5 µM, 10 µM, 20 µM, 40 µM, 80 µM, and 100 μM) for 24 h and 48 h using MTT [3-(4,5-dimethythiazol-2-yl)-2,5-diphenyl tetrazolium bromide] assays. The results suggested that doses of isoviolanthin up to 100 μM had no cytotoxic effect on normal LO2 cells, which maintained greater than 90% cell viability at 24 h (Figure 2A). Meanwhile, low concentrations of isoviolanthin (≤10 µM) did not significantly inhibit HCC cell viability, whereas higher doses of isoviolanthin (>10 µM) significantly suppressed HCC cell viability in a dose- and time-dependent manner (Figure 2B). Therefore, isoviolanthin levels (2.5 µM, 5 µM, and 10 µM) nontoxic to HCC cells were selected for subsequent studies.

It has been reported that treatment of HepG2 and Bel-7402 cells with 10 ng/mL TGF-β1 successfully promoted EMT [29]. To investigate the effects of isoviolanthin on the clonogenic potential of HepG2 and Bel-7402 cells, colony formation assay was performed. The results suggested that TGF-β1-treated HCC cells had significantly increased colony amounts over the untreated cells, while isoviolanthin (2.5 µM, 5 µM, and 10 µM) treatment dose-dependently inhibited the clonogenic ability of TGF-β1-treated HCC cells (Figure 2C, *p* < 0.05).

### 2.3. Isoviolanthin Inhibited the Transforming Growth Factor (TGF)-β1-Induced Migration and Invasion of HCC Cells

**Figure 3:**
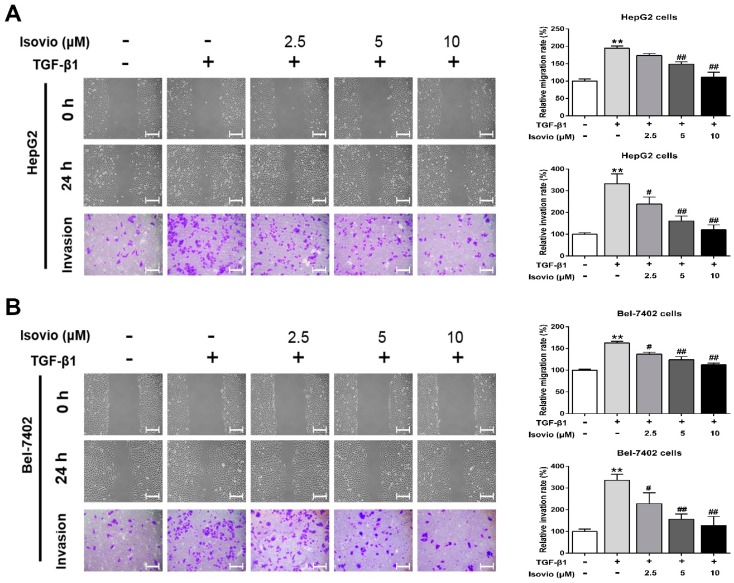
Effects of isoviolanthin on TGF-β1-induced migration and invasion of HCC cells. (**A**) HepG2 and (**B**) Bel-7402 cells were pretreated with 10 ng/mL TGF-β1 for 48 h, and then treated with isoviolanthin (2.5 µM, 5 µM, and 10 µM) for the last 24 h. The migration and invasion of HepG2 and Bel-7402 cells were measured by wound healing and transwell assays. Scale bars: 100 µm. Representative images and bar graphs (mean ± SD) are shown, *n* = 3. The magnification is ×100. ** *p* < 0.01 versus the control group; ^#^ *p* < 0.05, ^##^ *p* < 0.01 versus the TGF-β1 group.

Accumulating evidence also suggests that highly metastatic (invasive and motile) behaviors are the basic characteristics of EMT [30,31]. Herein, we performed wound healing and transwell assays to determine whether isoviolanthin inhibits the TGF-β1-induced migration and invasion of HCC cells. Our results revealed that the invasive and migratory potential of TGF-β1-treated HCC cells was remarkably higher than that of control cells at 24 h, while isoviolanthin (2.5 µM, 5 µM, and 10 µM) obviously attenuated TGF-β1-induced HCC cell migration and invasion in a dose-dependent manner (Figure 3A,B, *p* < 0.05). These results suggested that treatment with isoviolanthin effectively inhibited the TGF-β1-induced migration and invasion of HepG2 and Bel-7402 cells.

### 2.4. Isoviolanthin Inhibited TGF-β1-Induced MMP-2 and -9 in HCC Cells

**Figure 4:**
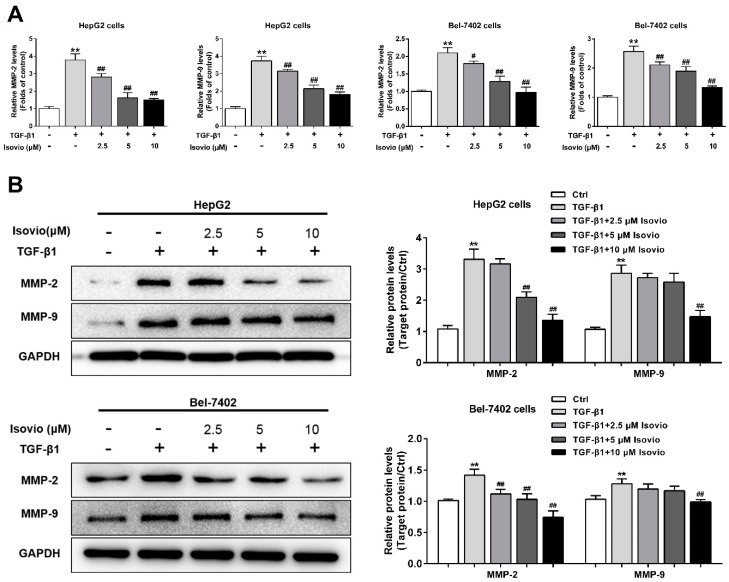
Effects of isoviolanthin on TGF-β1-induced matrix metalloproteinase (MMP)-2 and -9 in HCC cells. (**A**) HepG2 and Bel-7402 cells were treated with 10 ng/mL TGF-β1 for 48 h, during which isoviolanthin (2.5 µM, 5 µM, and 10 µM) was added for the last 24 h. The secretion of MMP-2 and -9 in HCC cell supernatants was detected by ELISA; (**B**) The protein levels of MMP-2 and -9 in HCC cells were examined by Western blot analysis. GAPDH was used as a loading control. Representative images and bar graphs (mean ± SD) are shown, *n* = 3. ** *p* < 0.01 versus the control group; ^#^ *p* < 0.05, ^##^ *p* < 0.01 versus the TGF-β1 group.

Since MMP-2 and -9 exert a critical role in the invasion and migration of HCC cells, the secretion of MMP-2 and -9 in the HCC cell supernatant was analyzed by ELISA. MMP-2 and -9 secretion were remarkably increased by TGF-β1, which could be dose-dependently alleviated by isoviolanthin (Figure 4A). Furthermore, Western blotting was used to detect the protein levels of MMP-2 and -9 in HCC cells. We found that isoviolanthin (2.5 µM, 5 µM, and 10 µM) significantly inhibited the upregulation of MMP-2 and -9 induced by TGF-β1 in a dose-dependent manner (Figure 4B). These results indicated that downregulation of MMP-2 and -9 might be involved in the anti-EMT activity of isoviolanthin.

### 2.5. Isoviolanthin Reversed the Induction of EMT Biomarkers by TGF-β1 in HCC Cells

**Figure 5:**
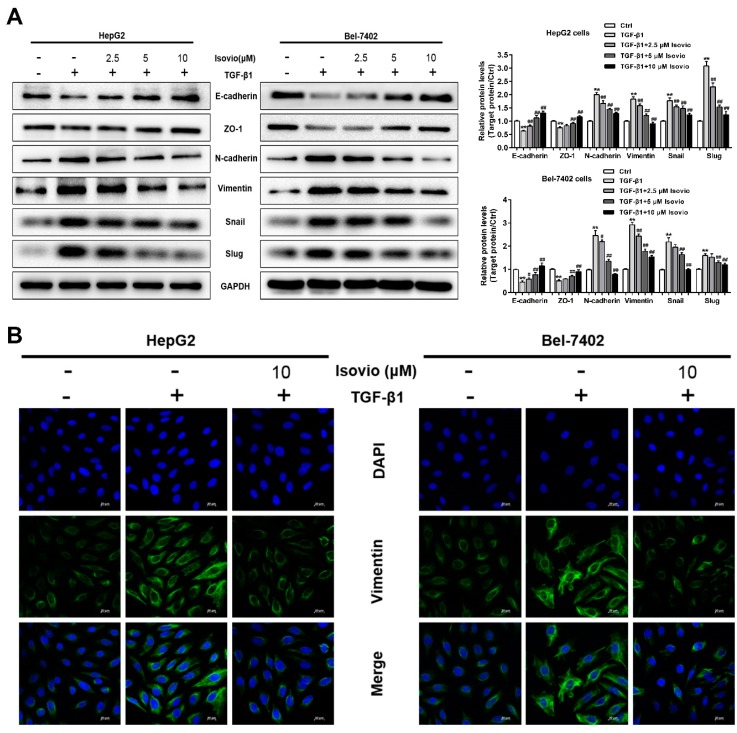
Effects of isoviolanthin on TGF-β1-induced epithelial–mesenchymal transition (EMT) in HCC cells. (**A**) HepG2 and Bel-7402 cells were treated with 10 ng/mL TGF-β1 for 48 h, during which the indicated concentrations of isoviolanthin were added for the last 24 h. The expression of the EMT markers E-cadherin, ZO-1, N-cadherin, vimentin, Snail, and Slug was assessed by Western blotting. GAPDH was used as a loading control; (**B**) E-cadherin and vimentin expression in HCC cells was determined by confocal microscopy. *Green fluorescence* indicates E-cadherin- and vimentin-positive expression, and *blue fluorescence* indicates 4’,6-diamidino-2-phenylindole (DAPI)-labeled nuclei. Scale bars: 20 µm. Representative images and bar graphs (mean ± SD) are shown, *n* = 3. ** *p* < 0.01 versus the control group; ^#^ *p* < 0.05, ^##^ *p* < 0.01 versus the TGF-β1 group.

To ascertain whether isoviolanthin plays a critical role in TGF-β1-induced EMT in HCC cells, Western blot was used to analyze EMT-associated biomarkers in HCC cells. The TGF-β1-induced expression of N-cadherin, vimentin, Snail, and Slug was significantly inhibited by isoviolanthin (2.5 µM, 5 µM, and 10 µM), whereas the expression of E-cadherin and ZO-1, which was inhibited by TGF-β1, was obviously increased in a dose-dependent manner (Figure 5A). In addition, we detected the expression of the mesenchymal marker vimentin of HCC cells by immunofluorescence staining, which revealed that TGF-β1 obviously elevated the vimentin expression in HepG2 and Bel-7402 cells. Compared with the TGF-β1-treated HCC cells, 10 µM isoviolanthin significantly decreased vimentin expression (Figure 5B). These results confirmed that isoviolanthin could effectively reverse the TGF-β1-induced changes in the expression of EMT biomarkers in HCC cells.

### 2.6. Isoviolanthin Inhibited TGF-β1-Induced EMT in HCC Cells via Regulating the TGF-β/Smad and PI3K/Akt/mTOR Pathways

**Figure 6:**
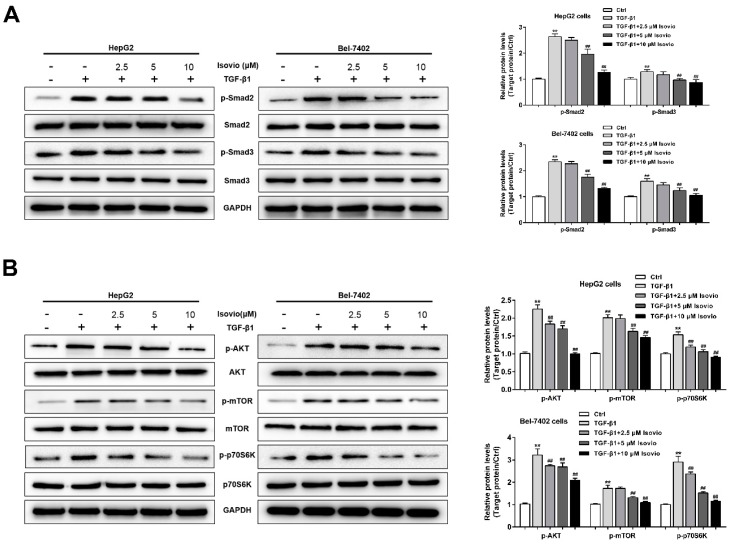
Effects of isoviolanthin on the TGF-β/Smad and PI3K/Akt/mTOR pathways in HCC cells. (**A**) HepG2 and Bel-7402 cells were treated with 10 ng/mL TGF-β1 for 48 h, during which the indicated concentrations of isoviolanthin were added for the last 24 h. The expression of TGF-β/Smad pathway-related proteins, such as Smad2, p-Smad2, Smad3, and p-Smad3, was examined by Western blot analysis; (**B**) The expression of PI3K/Akt/mTOR-pathway-related proteins, including Akt, p-Akt, mTOR, p-mTOR, P70S6K, and p-P70S6K, was examined by Western blot analysis. GAPDH was used as a loading control. Representative images and bar graphs (mean ± SD) are shown, *n* = 3. ** *p* < 0.01 versus the control group; ^##^ *p* < 0.01 versus the TGF-β1 group.

To explore the possible anti-EMT mechanism of isoviolanthin in TGF-β1-treated HCC cells, we examined the expression levels of proteins in TGF-β1-stimulated intracellular pathways. TGF-β has been shown to activate the canonical Smad pathway and PI3K/Akt/mTOR pathways [14,19,20]. In this study, Western blotting analyses showed that 10 ng/mL TGF-β1 strongly increased the phosphorylation of Smad2, Smad3, Akt, mTOR, and P70S6K (downstream of mTOR) in HCC cells, whereas total Smad2/3, Akt, mTOR, and P70S6K levels were unchanged. However, isoviolanthin (2.5 µM, 5 µM, and 10 µM) treatment dramatically decreased the upregulation of p-Smad2, p-Smad3, p-Akt, p-mTOR, and p-P70S6K levels by TGF-β1 in a dose-dependent manner (Figure 6A,B).

**Figure 7:**
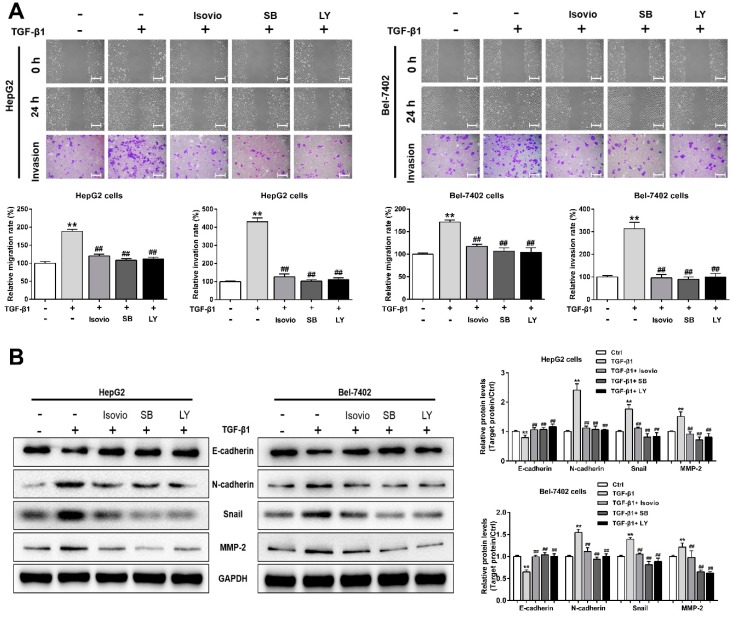
Verification that isoviolanthin affects TGF-β1-induced EMT, migration, and invasion in HCC cells by regulating the TGF-β/Smad and PI3K/Akt/mTOR pathways. (**A**) HepG2 and Bel-7402 cells were pretreated with 10 ng/mL TGF-β1 for 48 h and then treated with 10 µM isoviolanthin, 20 μM SB431542 (SB, TGF-β/Smad inhibitor), or 20 µM LY294002 (LY, PI3K/Akt inhibitor) for the last 24 h. HCC cell migration and invasion were measured by wound healing and transwell assays. Scale bars: 100 µm; (**B**) E-cadherin, N-cadherin, Snail, and MMP-2 expression in HCC cells was examined by Western blot analysis. GAPDH was used as a loading control. Representative images and bar graphs (mean ± SD) are shown, *n* = 3. ** *p* < 0.01 versus the control group; ^##^ *p* < 0.01 versus the TGF-β1 group.

To further investigate the functions of the TGF-β/Smad and PI3K/Akt/mTOR pathways in TGF-β1-induced EMT in HCC cells, these cells were treated with isoviolanthin (20 µM), the TGF-β/Smad inhibitor SB431542 (20 μM), or the PI3K/Akt inhibitor LY294002 (20 µM). Compared to the group treated with TGF-β1 alone, those treated with isoviolanthin, SB431542, or LY294002 showed markedly suppressed migration and invasion capacities (Figure 7A, *p* < 0.05). Furthermore, Western blot confirmed that the effects of SB431542 and LY294002 were consistent with those of isoviolanthin, as evidenced by the repression of N-cadherin, Snail, and MMP-2 expression levels and the induction of E-cadherin expression compared to the TGF-β1-treated group (Figure 7B). These results suggested that isoviolanthin suppressed TGF-β1-induced EMT through inhibition of the TGF-β/Smad and PI3K/Akt/mTOR pathways in HCC cells.

## 3. Discussion

Hepatocellular carcinoma (HCC) continues to result in extremely malignant tumors and is now the third-leading cause of cancer death worldwide. The tendency of HCC to metastasize is one of the important reasons for poor patient prognosis, and metastatic HCC is responsible for the majority of patient deaths [32,33]. Accumulating evidence suggests that epithelial–mesenchymal transition (EMT) is an important step during the progression and metastasis of HCC, and many studies have proven that 10 ng/mL TGF-β1 successfully promotes EMT via the induction of Snail, ZEB1, and other transcription factors in HCC cells [27,34,35,36]. Thus, the development of novel, nontoxic antimetastasis agents targeting EMT-related pathways has significant potential to benefit patients with metastatic HCC.

Although isoviolanthin has been identified and isolated from various medicinal herbs, its pharmacological effects have been rarely reported. Given that *Dendrobium officinale* extracts and polysaccharides have been shown to possess anticancer activities [21,23,37], we wondered whether flavonoid glycoside components of *Dendrobium officinale*, such as isoviolanthin, have antimetastasis effects on TGF-β1-treated HCC cells. In the current study, the MTT results showed that isoviolanthin was not toxic to normal hepatic LO2 cells at concentrations up to 100 µM, whereas isoviolanthin remarkably inhibited HepG2 and Bel-7402 cell proliferation in a dose- and time-dependent manner. Moreover, isoviolanthin obviously inhibited the clonogenic formation ability of TGF-β1-treated HCC cells in a dose-dependent manner.

TGF-β1 is a potent inducer of EMT that can abrogate cell–cell adhesion and promote mesenchymal phenotypes and migratory and invasive capabilities of HCC cells [38,39]. Thus, we explored the effect of isoviolanthin on the migration and invasion of TGF-β1-treated HCC cells, and wound healing and transwell analyses showed that isoviolanthin obviously suppressed the TGF-β1-induced migration and invasion of HCC cells in a dose-dependent manner. In addition, ELISA and Western blot analysis indicated that upregulation of MMP-2 and -9 induced by TGF-β1 was attenuated by isoviolanthin in a dose-dependent manner. Subsequently, we investigated the molecular mechanisms of the antimetastasis effects of isoviolanthin related to TGF-β1-induced EMT in HCC cells. It is well documented that the PI3K/Akt/mTOR and TGF-β/Smad signaling pathways are becoming increasingly difficult to ignore in investigations of EMT-related mechanisms in cancer cells [40,41,42,43]. TGF-β1-induced EMT contributes to the phosphorylation of Smad2/3, Akt, and mTOR, leading to the upregulation of EMT-related transcription factors (Snail, Slug) and mesenchymal markers (N-cadherin, vimentin) and the downregulation of epithelial makers (E-cadherin, ZO-1). We assessed the influence of isoviolanthin on EMT by immunofluorescence staining and Western blot analyses. Our results indicated that TGF-β1 upregulated the expression levels of epithelial makers and decreased those of mesenchymal markers and transcriptional factors, as well as activating the TGF-β/Smad and Akt/mTOR/P70S6K pathways. However, treatment with isoviolanthin significantly decreased TGF-β1-mediated Smad2/3, Akt, mTOR, and P70S6K phosphorylation and reversed EMT in a dose-dependent manner.

**Figure 8:**
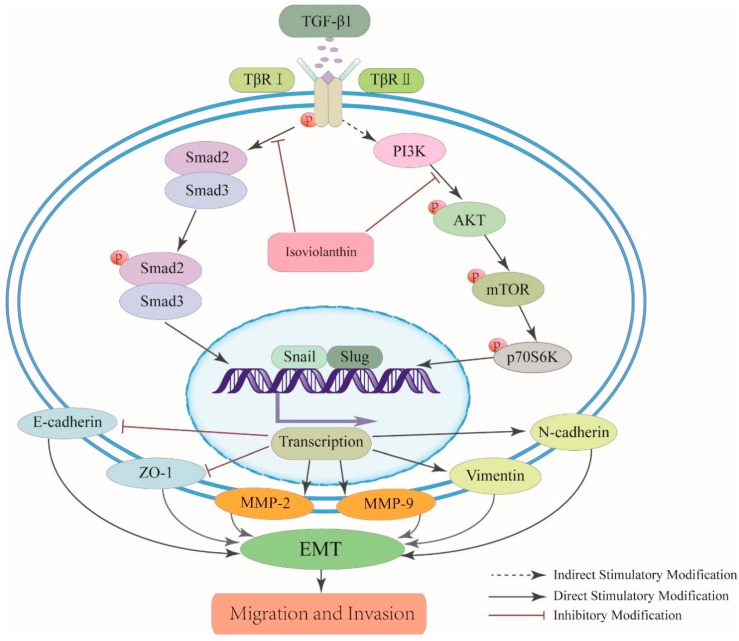
Schematic representation of the effect of isoviolanthin on TGF-β1-mediated EMT in HCC cells. Isoviolanthin inhibited the TGF-β/Smad and PI3K/Akt/mTOR pathways and finally suppressed HCC cell migration and invasion. “↓” indicates promotion; “⊥” indicates inhibition.

The relationship between EMT and inhibition of the TGF-β/Smad and Akt/mTOR/P70S6K pathways in HCC cells was further explored in this study. The PI3K/Akt inhibitor LY294002 and the TGF-β/Smad inhibitor SB431542 were used to confirm that the suppressive effect of isoviolanthin on EMT in HCC cells involves regulation of the TGF-β/Smad and PI3K/Akt/mTOR pathways, as evidenced by the downregulation of N-cadherin and Snail and the upregulation of E-cadherin compared to TGF-β1; these results were consistent with those obtained with isoviolanthin. Thus, we suggest that isoviolanthin deactivates the TGF-β/Smad and PI3K/Akt/mTOR pathways, thereby inhibiting EMT progression (Figure 8).

Taken together, these results suggest that isoviolanthin has anticancer effects on migration and invasion via regulating EMT in TGF-β1-treated HCC cells. Given that isoviolanthin has no cytotoxic effects on normal hepatic LO2 cells, we conclude that it may be suitable in combination with current chemotherapeutics, such as cisplatin, for the treatment of HCC; this possibility will be explored in our following study.

## 4. Materials and Methods

### 4.1. Plant Material and Chemicals

*Dendrobium officinale* plants were collected in the Zhejiang Province of China (Supplementary Materials Figure S3) as we described previously [44]. The purity of isoviolanthin was greater than 98% as determined by HPLC (C_27_H_30_O_14_, Figure 1A). Tween-20 and DMSO were purchased from Sigma (St. Louis, MO, USA). Petroleum ether, ethanol, *n*-butyl alcohol, and other reagents were of analytical grade.

### 4.2. Extraction of Crude Isoviolanthin

The *Dendrobium officinale* leaves were collected and cut into pieces, dried in an oven at 60 °C, and crushed into a powder. After 981.4 g of powder was weighed precisely, 80% ethanol was added, and extractions were performed in a reflux instrument for 2 h; the extraction procedure was repeated 3 times. The extract was filtered through gauze and concentrated by a rotary evaporator until the liquid become a thick paste. An aliquot of the thick paste was collected, and an adequate amount of distilled water was added to make a suspension, which was transferred into a separating funnel. Two volumes of petroleum ether (60–90 °C) were added into the suspension for the extraction. The upper layer was collected to obtain the petroleum ether fraction. In addition, 2 volumes of ethyl acetate were added to the lower layer, and the upper layer was collected to obtain the ethyl acetate fraction. Two volumes of n-butyl alcohol were added to the lower layer, the upper layer was collected as the n-butyl alcohol fraction, and the lower layer was collected as the water phase fraction. The n-butyl alcohol fraction was collected, concentrated, and dried in a vacuum drier; 41.1 g of n-butyl alcohol extract was obtained.

### 4.3. Purification of Isoviolanthin

The n-butyl alcohol fraction was purified using an AB-8 macroporous absorption resin (Beijing Manchang Technology Co., Ltd., Beijing, China). The procedure was as follows. An adequate amount of the n-butyl alcohol fraction was dissolved in 1 volume of distilled water by ultrasonication for 15 min to obtain a suspension. Then, 3 kg of AB-8 macroporous absorption resin was collected, and 95% ethanol was added to the resin, which was then loaded into a 10 × 100 cm chromatographic column. The elution phase was 95% ethanol, which was loaded into the column to remove impurities and to activate the column. In addition, distilled water was loaded as the elution phase to elute all the ethanol. The flow rate was 1 BV/h. The suspension was loaded into the column and eluted with distilled water, 10% ethanol, 30% ethanol, 40% ethanol, and 95% ethanol. In total, 5–7 BVs were collected in each elution phase. The target compounds were all eluted in 30% ethanol. The flavonoid content in each elution fraction was detected by HPLC (LC-20AT, Shimadzu, Japan) at a wavelength of 270 nm. We selected the elution fractions that contained the most flavonoids for further isolation and analysis. The 30% ethanol fraction was collected, dried by a vacuum drier, and weighed. Eventually, 5.1 g of residue was obtained.

The 30% ethanol fraction obtained above was further purified using a Sephadex LH-20 (Solarbio, Beijing, China). An adequate amount of the n-butyl alcohol fraction was dissolved in 50% methanol by mixing. The mixture was filtered through a 0.45 μm filter for purification. The appropriate Sephadex LH-20 resin was soaked in 50% methanol overnight to allow it to swell and then loaded in a 2.5 × 100 cm column. The column was eluted with 50% methanol at an elution speed of 1 mL/min. The mixture was loaded and eluted in 50% methanol. The elution phase was collected and concentrated. The purification procedure was repeated with a 1 × 100 cm column. Finally, a purified fraction in 50% methanol was obtained.

The 50% methanol fraction was purified using Pre-HPLC (LC-8A, Shimadzu, Japan). The chromatographic conditions were as follows: XBridgeC18, OD column (Waters, Milford, MA, USA); elution speed, 2.5 mL/min; detection wavelength, 270 nm; and elution phase, 20% methanol. Pre-HPLC was performed with a gradient elution phase. The elution phase changed from 20% to 30%, 50%, and 95% methanol. The purification procedure was repeated several times, and isoviolanthin was obtained.

### 4.4. Structural Analysis of Isoviolanthin

An adequate amount of the above isolated compound was dissolved in methanol and water to test its solubility. The methanol solution was processed for flavonoid characterization. First, magnesite powder and HCl were added into the methanol solution to test whether the compound was a flavonoid. Then, the solution was further evaluated by the Molisch reaction to determine whether it contained sugar. Then, the purity of the isolated compound was determined by HPLC. The structure was further analyzed by UV, ESIMS (Thermo Finnigan LCQ FLEET, Riviera Beach, FL, USA), ^1^H-NMR, and ^13^C-NMR (Avance III HD 400 MHz Digital NMR Spectrometer, Bruker, Karlsruhe, Germany).

### 4.5. Cell Culture and Treatment

Two human hepatocellular carcinoma cell lines—HepG2 and Bel-7402 cells—and the human L02 normal liver cell line were kind gifts from Dr. Biaoyan Du, Professor, Guangzhou University of Chinese Medicine, Guangzhou, China. These cell lines were propagated in high-glucose Dulbecco’s Modified Eagle’s Medium (DMEM) supplemented with 10% fetal bovine serum (FBS), 0.5% streptomycin, and penicillin at 37 °C in 5% CO_2_. The media components were obtained from Gibco (Grand Island, NY, USA). HepG2 and Bel-7402 cells were incubated with 10 ng/mL TGF-β1 (PeproTech, Rocky Hill, NJ, USA) to induce EMT as reported previously [29].

### 4.6. Influence of Isoviolanthin on Cell Proliferation

HepG2, Bel-7402, and L02 cells (2.5 × 10^3^/well) were seeded in 96-well plates and incubated overnight in an incubator. Cells were treated with various concentrations of isoviolanthin (2.5 µM, 5 µM, 10 µM, 20 µM, 40 µM, 80 µM, and 100 μM) for 24 h and 48 h. After 100 μL of MTT reagent (0.5 mg/mL, Sigma-Aldrich, St. Louis, MO, USA) was added for 4 h, each well was dissolved with 100 μL of DMSO, the plate was shaken for 10 min, and the absorbance at 490 nm was measured using a microplate reader (Bio-Rad, Hercules, CA, USA).

### 4.7. ELISA

After pretreatment with TGF-β1 for 48 h and incubation with isoviolanthin (2.5 µM, 5 µM, and 10 µM) for the last 24 h, the HepG2 and Bel-7402 cell culture supernatants were collected. The levels of MMP-2 and MMP-9 in HepG2 and Bel-7402 cell culture supernatants were detected using human matrix metalloproteinase (MMP)-2 and -9 ELISA kits (ABclonal, Wuhan, China) according to the manufacturer’s instructions.

### 4.8. Colony Forming Assay

HepG2 and Bel-7402 cells (1 × 10^3^ cells/ well) were seeded in 6-well plates, and cotreated with isoviolanthin (2.5 µM, 5 µM, and 10 µM) and TGF-β1 (10 ng/mL) for two weeks. Then, the cells were fixed with 4% paraformaldehyde and stained with 0.1% crystal violet. The plates were photographed and the colonies were counted by Image-Pro Plus 6.0 software (Bethesda, MD, USA).

### 4.9. Immunofluorescence Analysis

HepG2 and Bel-7402 cells (2 × 10^4^ cells/well) in 4-well chamber slides were treated with 10 ng/mL TGF-β1 for 48 h, and isoviolanthin (10 µM) was added for the last 24 h. Cells were fixed in 4% formaldehyde for 15 min, blocked with 5% bovine serum albumin (BSA) and 0.5% Triton X-100 for 2 h at 37 °C, and then incubated overnight at 4 °C with anti-vimentin antibody (Cell Signaling Technology, Danvers, MA, USA, 1:100). Cells were then incubated with secondary Fluorescein isothiocyanate (FITC)-conjugated anti-rabbit IgG antibody (Cell Signaling Technology, 1:1000) for 1 h and visualized by incubation with 4’,6-diamidino-2-phenylindole (DAPI) (Beyotime, Guangzhou, China) for 15 min at room temperature. Fluorescence was captured using laser confocal microscopy 880 with Airyscan (Zeiss, Jena, Germany).

### 4.10. Wound Healing and Transwell Assays

HepG2 and Bel-7402 cells (3 × 10^5^ cells/well) were pretreated with 10 ng/mL TGF-β1 for 24 h in 6-well plates, the center of each well was gently scraped with a 200 μL pipette tip, and the wells were washed three times with PBS. Then, cells were cotreated with isoviolanthin (2.5 µM, 5 µM, and 10 µM) and TGF-β1 (10 ng/mL) for 24 h, and images of cell migration were obtained at 0 and 24 h using an inverted microscope (Olympus, Hamburg, Germany). For transwell assays, after treatment with 10 ng/mL TGF-β1 for 24 h, cells (2 × 10^4^ cells/well) in 200 µL of serum-free DMEM containing isoviolanthin (2.5 µM, 5 µM, and 10 µM) were cultured in the upper chambers (8 μm pore size, Invitrogen, Carlsbad, CA, USA), while 800 µL of DMEM (15% FBS) containing 10 ng/mL TGF-β1 was added to the lower chambers. After a 24 h incubation, invaded cells were fixed with 4% paraformaldehyde for 15 min and stained with 0.1% crystal violet for 20 min. Images of cells that had invaded the Matrigel-coated filter were taken, and the number of invaded cells was counted by Image-Pro Plus 6.0 software (Bethesda, MD, USA).

#### 4.11. Western Blot Analysis

HepG2 and Bel-7402 cells (2.5 × 10^5^ cells/well) were pretreated with 10 ng/mL TGF-β1 for 48 h in 100 mm tissue culture dishes, followed by treatment with isoviolanthin (2.5 µM, 5 µM, and 10 µM), LY294002 (20 µM, MedChem Express, NJ, USA), or SB431542 (20 µM, MedChem Express) for the last 24 h. Total protein was isolated with radioimmunoprecipitation assay buffer (RIPA buffer) mixed 1% PMSF (Phenylmethanesulfony fluoride) as we described previously [45], and 30 micrograms of protein was separated by 8–12% SDS-PAGE and transferred to PVDF membranes (Millipore, Bedford, MA, USA). Membranes were blocked with 5% nonfat dry milk in tris-buffered saline (TBS)-T (0.1% Tween-20) for 2 h at room temperature and then incubated overnight at 4 °C with primary antibodies against E-cadherin, glyceraldehyde 3-phosphate dehydrogenase (GAPDH), ZO-1, N-cadherin, vimentin, Snail, Slug, mTOR, p-mTOR, P70S6K, and p-P70S6K (1:1000, Cell Signaling Technologies, Beverly, MA, USA); and Smad2, p-Smad2, MMP-2, MMP-9, Smad3, Akt, p-Akt, and p-Smad3 (1:1000, ABclonal, Wuhan, China). Blots were incubated for 2 h at room temperature with the corresponding secondary horseradish peroxidase (HRP)-conjugated goat anti-rabbit or anti-mouse IgG antibodies (1: 3000, ABclonal), and the band densities were visualized with enhanced chemiluminescence HRP substrate (Millipore, Bedford, MA, USA) and a Tanon detection system (Shanghai, China).

### 4.12. Statistical Analysis

The data of MS were analyzed with the Finnigan Xcalibur 2.0 advanced chromatography workstation (Thermo Quest Corporation, San Jose, CA, USA). Data were statistical analyzed with SPSS 18.0 (SPSS Inc., Chicago, IL, USA), multiple comparisons were performed using one-way ANOVA, and data are presented as the mean ± SD with GraphPad Prism 6.0 (GraphPad Software, CA, USA). A value of *p* < 0.05 indicated statistical significance. 

## 5. Conclusions

Isoviolanthin may be one of the most important components responsible for the antimetastasis activity of *Dendrobium officinale*. For the first time, we provide evidence that isoviolanthin targets the TGF-β/Smad and PI3K/Akt/mTOR pathways to repress TGF-β1-induced EMT phenotypes in HepG2 and Bel-7402 HCC cells. Furthermore, these results confirm that isoviolanthin could be a promising natural compound with low toxicity for the treatment of metastatic HCC by affecting TGF-β1-induced EMT.

## Supplementary Materials

Supplementary materials can be found at http://www.mdpi.com/1422-0067/19/6/1556/s1.

## Abbreviations

UV	Ultraviolet Radiation
HPLC	High-Performance Liquid Chromatography
Pre-HPLC	Preparative High-Performance Liquid Chromatography
LC-MS	Liquid Chromatography-Mass spectrometry
NMR	Nuclear Magnetic Resonance
HCC	Hepatocellular Carcinoma
TGF-β1	Transforming Growth Factor-β1
EMT	Epithelial–Mesenchymal Transition
MMPs	Matrix Metalloproteinases
PI3K	Phosphatidylinositol-3-Kinase
Akt	Protein Kinase B
TGF-β1	Transforming Growth Factor-β1
P70S6K	p70 Ribosomal Protein S6 Kinase
mTOR	Mammalian Target of Rapamycin
