# Supplementary material for: Isoviolanthin Extracted from Dendrobium officinale Reverses TGF-β1-Mediated Epithelial–Mesenchymal Transition in Hepatocellular Carcinoma Cells via Deactivating the TGF-β/Smad and PI3K/Akt/mTOR Signaling Pathways
Source: Int J Mol Sci. 2018 May 23;19(6):1556. doi: 10.3390/ijms19061556 (PMC6032198; doi:10.3390/ijms19061556)
Supplement: Supplementary file 1 [file ijms-19-01556-s001.zip › ijms-293853-supplementary.docx]

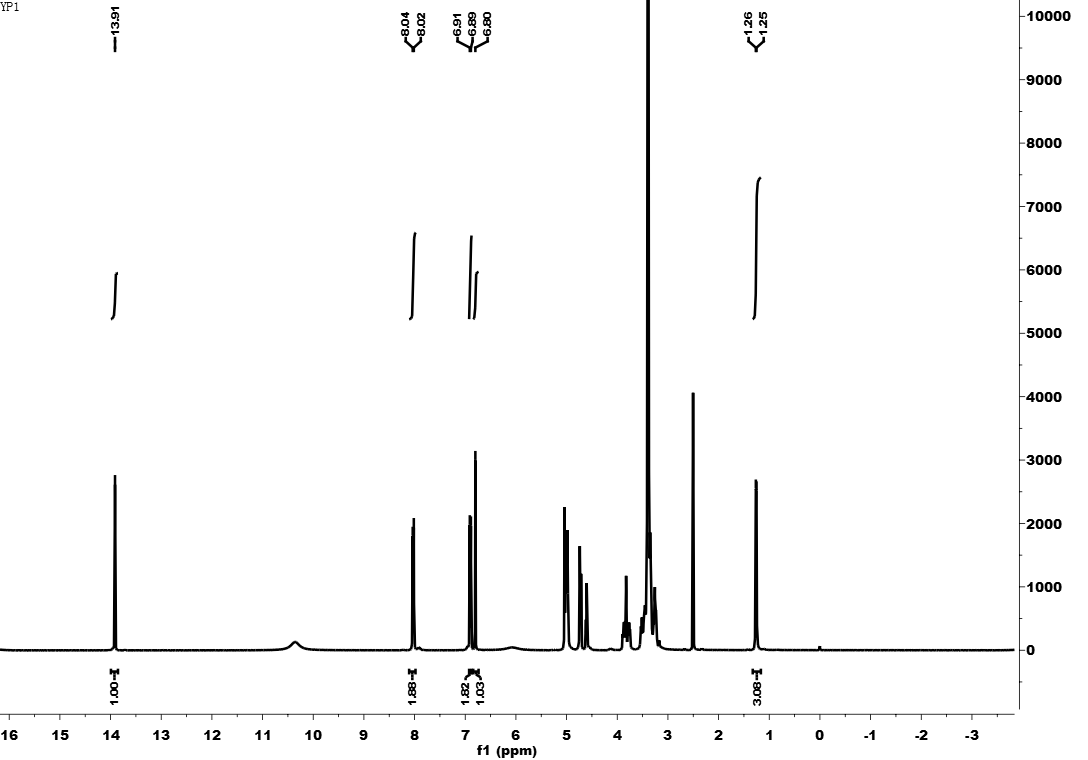


**Figure S1.** The ^1^H-NMR spectra of isoviolanthin.


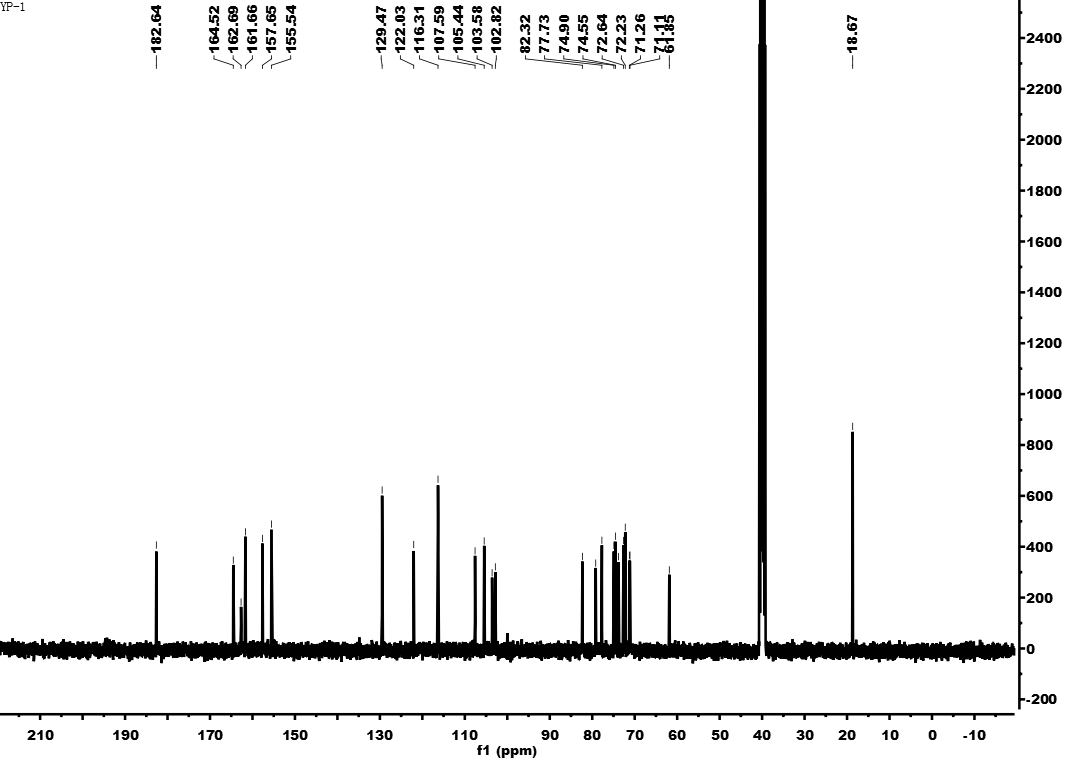


**Figure S2.** The ^13^C-NMR spectra of isoviolanthin.


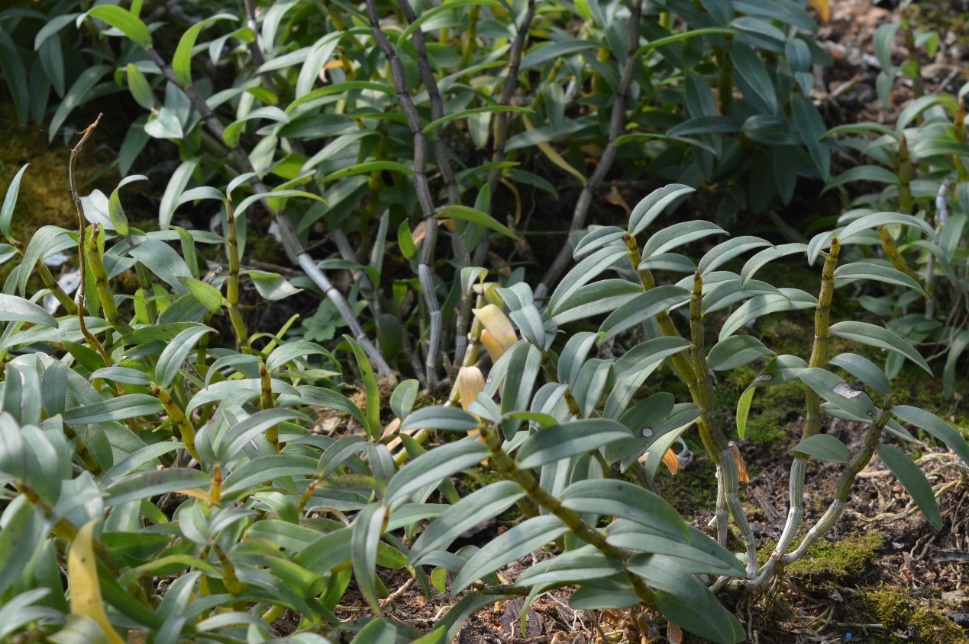


**Figure S3.** Images of *Dendrobium officinale* and its stems.
